# Lactic acid bacteria: beyond fermentation to bio-protection against fungal spoilage and mycotoxins in food systems

**DOI:** 10.3389/fmicb.2025.1580670

**Published:** 2025-06-30

**Authors:** Md Saifur Rahman, Samira Soltani, Gisèle LaPointe, Salwa Karboune, Ismail Fliss

**Affiliations:** ^1^Food Science Department, Food and Agriculture Faculty, Université Laval, Québec, QC, Canada; ^2^Saskatoon Research Centre, Agriculture and Agri-Food Canada, Saskatoon, SK, Canada; ^3^Department of Food Science, Canadian Research Institute for Food Safety, University of Guelph, Guelph, ON, Canada; ^4^Department of Food Science and Agricultural Chemistry, Macdonald Campus, McGill University, Montreal, QC, Canada; ^5^Institute of Nutrition and Functional Foods, Université Laval, Québec, QC, Canada

**Keywords:** antimicrobial, lactic acid bacteria (LAB), bio-preservation, shelf-life, fungal spoilage, mycotoxins

## Abstract

Recent outbreaks of foodborne diseases have highlighted the challenges of maintaining food safety, emphasizing the need for effective strategies to control pathogens and spoilage organisms. Toxins produced by indigenous fungi pose serious economic issues and undermine food security. Mycotoxin spoilage is a ubiquitous hazard that affects all food commodities; however, bakery products, dairy, fruits, vegetables, and meat are particularly vulnerable. The quality of food is perceived through senses such as taste, aroma, and texture. These sensory attributes significantly impact the overall sensation of the product and determine whether it will be accepted or rejected by consumers. Spoilage not only reduces consumer satisfaction but also drastically shortens the shelf life of food. This review highlights the ability of Lactic Acid Bacteria (LABs) to produce diverse antimicrobials, emphasizing antifungal metabolites as effective tools for enhancing food preservation and extending shelf life. As consumer demand for ‘clean label’ solutions increases, these natural antimicrobials promise safe and effective alternatives for enhancing food safety, reducing fungal spoilage, and extending shelf life of various perishable food commodities and reducing economic losses.

## Introduction

Fungi comprise more than 1 million species and, unlike plants, lack chlorophyll, relying instead on external sources of organic matter. They thrive in damp, dark environments (Moore et al., 2020). Mold, a common fungal form, frequently contaminates food due to their widespread distribution in nature and adaptability to various conditions. Contamination often occurs in food processing facilities via raw materials, surfaces, and equipment. In unhygienic environments, fungal spores can travel through air and adhere to clothing or footwear, potentially introducing pollutants into production areas. Fungal contamination negatively impacts the quality, safety, and longevity of feed and food commodities, causing significant economic losses. Studies indicate that fungal spoilage accounts for approximately 5–10% of global food production losses and 50% of fruit and vegetable waste in tropical regions (Mastanjević et al., 2022). Australia alone incurs around $10 million annually in food losses attributed to fungal spoilage (Cheong et al., 2014), while bread spoilage costs in Western Europe exceed €200 million annually (Abdelhameed and Khalifa, 2024). Crop contamination by fungi such as *Aspergillus* and *Fusarium* also results in agricultural losses of up to $60 billion per year.

In addition to spoilage, some fungi can pose serious risks to food and feed safety through the production of mycotoxins, which are harmful secondary metabolites produced by certain filamentous fungi, particularly from the genera *Aspergillus*, *Penicillium*, and *Fusarium*. Major mycotoxins include ochratoxins, fumonisins, and aflatoxins, which have been linked to liver cancer, nephropathy, immunosuppression, and growth impairment in both humans and animals (Bryden, 2007; Milićević et al., 2016). Aflatoxins, which contaminate food consumed by an estimated 4.5 billion individuals in less economically developed nations, can cause acute poisoning resulting in death in approximately 40% of cases, as documented in Kenya (CDC, 2004). Aflatoxin B1 (AFB1), produced mainly by *Aspergillus flavus* and *Aspergillus parasiticus*, is the most prevalent form. Other types such as G1 and M2 are found in grains and dairy products, respectively (Min et al., 2021). The World Health Organization (WHO) has classified aflatoxins as Group 1 human carcinogenic (Kensler et al., 2011).

Beyond aflatoxins, other foodborne molds also produce toxic secondary metabolites that persist even after processing (Bullerman and Bianchini, 2007), posing chronic risks to immunocompromised individuals (Milićević et al., 2010). The prevalence of fungal contamination and mycotoxin production in food and feed systems remains a global concern, with some estimates suggesting that up to 25% of the world’s food supply is affected, raising significant alarms among researchers, manufacturers, and regulatory bodies (Topping and Clifton, 2001). Notably, species such as *Candida* spp., *Fusarium* spp., and *Aspergillus* spp. have been identified as opportunistic pathogens capable of causing systemic infections in immunocompromised hosts.

Despite their detrimental effects, certain fungi and yeasts contribute positively to the food industry. They play important roles in traditional fermentation processes, improving organoleptic properties such as flavor, aroma, and texture, as well as enhancing nutritional value through the production of organic acids, enzymes, and other bioactive compounds (Pouris et al., 2024). Genera such as *Pichia, Geotrichum, Candida, Zygosaccharomyces, Kluyveromyces, Torulaspora, and Saccharomyces*, along with molds such as *Aspergillus, Penicillium, Geotrichum, Phoma, Mucor*, and *Rhizopus*, are frequently employed as stater cultures in the fermentation of cereals, meats, and milk (Rai and Jeyaram, 2017; Wei et al., 2019). Nonetheless, contamination by acid-tolerant fungi during fermentation remains a persistent risk (Dinakarkumar et al., 2024).

Chemical additives, such as potassium sorbate, sulfur dioxide, and calcium propionate, are commonly used to combat food spoilage by yeasts and molds. However, these additives fail to match the rising demand of consumers for “clean label” products made with natural ingredients (Leyva Salas et al., 2017). Alternative methods for detoxifying or removing mycotoxins from feed or food products include physical methods such as the use of ultraviolet light, ionizing radiation, or heat; chemical processes such as acid or alkaline-based methods; and hydrolytic, chlorinating, oxidizing, or reducing agents. However, some fungal strains and their mycotoxins are resistant to these methods, which can negatively impact product quality and increase processing costs (Molina-Hernandez et al., 2025). Therefore, innovative, natural, safe, and cost-effective solutions are required to address fungal contamination in food.

In this context, bio-preservation or the utilization of precisely identified microbes or their respective antimicrobial substances in food, has risen, driven by an expanding consumer preference for more natural preservation methods as opposed to synthetic chemicals (Pouris et al., 2024). Among these, lactic acid bacteria (LAB) have emerged as strong candidates for antifungal biopreservation. For decades, LAB has been utilized in conventional food fermentations and many species have been granted ‘Generally Recognized as Safe’ (GRAS) and ‘Qualified Presumption of Safety’ (QPS) certification through the European Food Safety Authority (EFSA) and the American Food and Drug Administration (FDA) (Leuschner et al., 2010). LAB are defined as Gram-positive, non-spore-forming rods or cocci, that ferment carbohydrates to produce lactic acid as their main metabolic end product. LAB are commonly classified into three physiological groups based on their fermentation pathways: obligate homofermentative, which produce lactic acid as the sole end-product from glucose; obligate heterofermentative, which convert glucose into lactic acid, ethanol or acetic acid, and CO₂; and facultative heterofermentative, which primarily produce lactic acid from glucose but can shift to heterofermentative pathways under specific conditions (Mokoena, 2017).

LAB strains have been referred to as “green preservatives” because they could suppress several undesirable microorganisms in food, including fungi, through the production of natural antimicrobial compounds. In the context of food safety, green preservatives refer to naturally derived or minimally processed substances that offer microbial control without the use of synthetic chemical additives, aligning with consumer demand for clean-label and safer food products (Pawlowska et al., 2012). Many LAB strains are known to produce a variety of antifungal substances including alcohols, lactones, aldehydes, diacetyl, organic acids, bioactive antimycotic peptides, hydrogen peroxide, carboxylic acids, fatty acids, and bacteriocins (Crowley et al., 2013). Recently, LAB strains have been used to control fungi in both fermented and non-fermented foods, including cereals, yogurt and fresh products (Aunsbjerg et al., 2015). In addition to inhibiting fungal growth, some LAB strains can bind and neutralize mycotoxins via interactions with their cell wall components.

However, several limitations affect the widespread application of LAB in food systems. Their antifungal activity is highly strain-specific and may vary depending on food matrix composition. Moreover, the stability of bioactive metabolites under processing and storage conditions poses a challenge. Regulatory constraints can also limit the use of novel LAB strains in food products, despite their recognized safety in traditional applications.

Although multiple scholarly reviews have described the antifungal properties of LAB, most focus on their metabolites diversity or general application without deeply exploring LAB-mycotoxin interactions or practical performance in real food matrices (Crowley et al., 2013; Liu A. et al., 2022; Nasrollahzadeh et al., 2022; Shi and Maktabdar, 2022) This review aims to bridge that gap by critically evaluating the mechanisms through which LAB detoxify mycotoxins, exploring LAB-fungi interactions in food systems, and identifying promising LAB strains for future bio-preservation applications in both fermented and non-fermented products.

This narrative review was based on a non-systematic literature search conducted primarily through ScienceDirect, PubMed, Scopus, and Google Scholar. Keywords included “lactic acid bacteria,” “mycotoxins,” “food spoilage fungi,” “bio-preservation,” and “antifungal metabolites.” Preference was given to peer-reviewed articles published between 2010 and 2024, with emphasis on studies from the last 5–7 years. Older but widely cited or foundational sources were included when recent data were unavailable.

### Fungi in food products

Fungi significantly contribute to enhancing the flavor of various food items because of their metabolic activities and the production of a wide range of aroma molecules, organic acids, and flavor-active compounds. On the other hand, some fungi cause defects that may be visible or invisible, such as undesirable odors and tastes, leading to substantial food waste and financial setbacks (Melini and Melini, 2024). Mitigating fungal spoilage is a significant challenge for industry professionals, and researchers are seeking effective methods to prevent and minimize fungal contamination of many types of food products (Mhlongo et al., 2019).

Climate change is increasingly recognized as a critical factor influencing the prevalence and distribution of mycotoxigenic fungi. Environmental stressors such as elevated temperatures, high humidity, drought followed by rehydration, and rising atmospheric CO₂ levels have been shown to affect fungal growth and mycotoxin production (Chhaya et al., 2022). These factors can alter gene expression related to toxin biosynthesis (Verheecke-Vaessen et al., 2019). For example, aflatoxin production by *A. flavus* influences under specific combinations of high temperature, water activity, and CO₂ concentration (Medina et al., 2014; Medina et al., 2013). Likewise, extreme rainfall increases deoxynivalenol (DON) accumulation in cereals at harvest (Franz et al., 2009), and elevated CO₂ has been associated with increased plant susceptibility to *Fusarium* infection (Vaughan et al., 2016). Such ecological shifts have facilitated the spread of aflatoxigenic species into temperate regions such as Central and Southern Europe (Kos et al., 2020), raising concerns about the adequacy of traditional storage and food safety practices under changing climatic conditions.

### Fungi in bakery products

Bread is particularly vulnerable to fungal contamination, as bakery products are perishable due to moisture loss and microbiological decomposition. Fungal spoilage is considered the leading cause of deterioration in these products, often surpassing bacterial spoilage in frequency and severity (Abdelhameed and Khalifa, 2024). Currently, weak organic acids such as calcium propionate are added to protect food matrices. However, in recent years, both legislators and consumers have advocated for the removal of preservatives from food products due to growing concerns about their potential health risks, including allergic reactions, gut microbiota disruption, and links to chronic diseases (Li et al., 2024). This demand has also been driven by the clean-label movement, which favors minimally processed products with recognizable, natural ingredients. Nonetheless, reducing the number of additives used to prevent mold spoilage in bakery items may, in most cases, decrease the shelf life of the product (Garcia et al., 2019a). In response, there is increasing interest in natural preservation strategies, including the use of antimicrobial metabolites produced by lactic acid bacteria (LAB), essential oils, plant extracts, and biopreservative packaging systems that aim to maintain product safety while meeting consumer expectations (Castellano et al., 2017). Molds in bread pose a significant economic issue, leading to 1–5% product losses depending on the season, product type, and processing technique (Kam et al., 2007). In Europe alone, economic losses due to fungal contamination in bread have been estimated at over €200 million per year, while in tropical regions, spoilage-related losses in baked goods may reach up to 11% (Garcia et al., 2019a). Several studies have identified various species involved in the mold spoilage of bread, with those belong to *Eurotium, Aspergillus*, and *Penicillium* being the most common and significant genera. *Cladosporium*, *Mucor*, and *Rhizopus* have also been found in bread products; however, their higher water activity (Aw) requirements, along with sensitivity to factors such as pH, temperature, and oxygen availability, make them less likely to contaminate bakery products under typical storage conditions (Garcia et al., 2021).

In addition to the economic losses associated with bread goods, mycotoxin production poses a potential health risk. The *Eurotium* species typically establishes itself as the primary fungus that infests inadequately stored or dried products. Their growth increases the water activity (Aw), facilitating the proliferation of a number of other species (e.g., *Penicillium* sp. and *Aspergillus* sp.). Given that *Eurotium* sp. does not generate substantial mycotoxin quantities, it is essential to comprehend how *Aspergillus* and *Penicillium* species can flourish and contaminate bread products, as some species are capable of doing so. Aflatoxicosis, caused by the ingestion of aflatoxins primarily produced by *A. flavus*, remains a significant public health concern, with over 500 reported cases and 200 deaths worldwide since 2004 due to contaminated food products, including bakery items (Shabeer et al., 2022). For instance, in the United States, toxigenic *A. flavus* was recovered from three out of the 15 home bakery products inspected, including a toxigenic *Penicillium* strain (species not specified) from wheat flour and bread (Girardin, 1997). Numerous *Penicillium* species, including *Penicillium chrysogenum*, produce mycotoxins (Garcia et al., 2021). Despite baking temperatures reaching 200–250°C, certain mycotoxins such as ochratoxin A and aflatoxins produced by *Aspergillus* and *Penicillium* spp. can persist due to their thermal stability (Garcia et al., 2019b; Kabak, 2009). OTA and aflatoxins, produced by *Aspergillus* and *Penicillium*, can remain active even after baking due to their thermal stability, posing long-term risks including immunosuppression and carcinogenicity (Gupta et al., 2022). The presence of such mycotoxins in flour or via post-processing contamination is a growing concern for consumer safety and regulatory compliance.

Post-baking yeast contamination is also a major contributor to spoilage. Since baking eliminates most microorganisms, contamination occurs during post-baking stages such as cooling, slicing, packaging, and storage (Vermelho et al., 2024). Airborne yeasts may originate from poorly sanitized equipment (e.g., slicers, conveyor belts, racks), packaging materials, or humid storage environments (Ali et al., 2023).

Evidence of superficial yeast growth on products indicates yeast spoilage (cream or white patches). *Hyphopichia burtonii* (formerly *Pichia burtonii*) often referred to as “chalk mold,” is a common spoilage yeast, often forming cream or white patches on bread prior to mold development. Its rapid proliferation and resistance to standard storage practices make it particularly problematic.

Preventive strategies focus on minimizing contamination routes. These include high-efficiency particulate air (HEPA) filtration in production areas, improved sanitation of equipment, and reduced manual handling. Modified-atmosphere packaging (MAP), incorporation of antifungal compounds derived from lactic acid bacteria (LAB), and control of relative humidity are effective methods to extend shelf life and suppress yeast growth (Garnier et al., 2017). In British bread, although filamentous fungi are more frequently detected in spoiled bread due to easier identification, spoilage yeasts such as *Hyp. Burtonii* remain a significant concern (Saranraj and Sivasakthivelan, 2015).

While molds are typically associated with spoilage in bakery products, certain fungal species have long played beneficial roles in traditional fermentations. For instance, *Aspergillus oryzae* is used industrially for enzyme production that can improve dough handling and baking performance, though it is more common in Asian food fermentations (Chang and Ehrlich, 2010; Machida et al., 2008). In bakery systems, the primary fermentative agents are yeasts and lactic acid bacteria (Erten et al., 2014). In addition to *Saccharomyces cerevisiae*, non-conventional yeasts such as *Candida milleri* (now *Kazachstania humilis*), *Kazachstania exigua*, *Torulaspora delbrueckii*, and *Wickerhamomyces anomalus* have been frequently isolated from sourdough and artisanal bakery environments (Ceresino et al., 2024). To manage spoilage without disrupting beneficial fermentative yeasts, selected LAB strains are increasingly used as natural preservatives, producing antifungal compounds that inhibit molds while supporting desirable sourdough microflora (Pérez-Alvarado et al., 2022). These yeasts contribute to dough acidification, enhanced aromatic complexity, and desirable textural properties. However, uncontrolled fungal growth remains a major concern.

Mycotoxins such as aflatoxins, ochratoxin A (OTA), and patulin, commonly associated with moldy grains and baked products, are linked to hepatotoxic, nephrotoxic, immunosuppressive, and carcinogenic effects. OTA, for example, is a potent nephrotoxin and possible human carcinogen (Group 2B, IARC) (Nazareth et al., 2024) The European Union has established maximum levels for OTA in cereals and cereal products at 3 μg/kg (European Commission, 2006). In the United States, although limits are less specific for bakery products, the FDA recommends limits for total aflatoxins in human food at 20 μg/kg (FDA, 2021).

### Fungi in dairy products

Milk and dairy products are known for their lower susceptibility to spoilage compared to other food items, such as fruits or vegetables, because of thermal treatment processes, such as pasteurization and subsequent refrigerated storage. Despite these protective measures, a significant number of yeast and mold species exhibit a remarkable ability to survive and thrive in these environments (Garnier et al., 2017). This resilience can be attributed to the remarkable adaptation capabilities of fungi, which enable them to utilize a wide range of substrates found in dairy products, including lipids, organic acids, carbohydrates, and proteins. Consequently, their presence can cause changes such as visible fungal growth, off-flavors, and odors, as well as alterations to the color and texture of the products (Bento de Carvalho et al., 2024).

Thus far, up to 100 mold species have been shown to contribute to dairy product deterioration (Garnier et al., 2017). *Penicillium* species are among the most prevalent, followed by *Aspergillus*, *Mucor* and various yeast genera (Garnier et al., 2017). These fungi are the main contaminants in dairy products, leading to considerable annual food waste and economic setbacks on a global scale (Garnier et al., 2017). For instance, industry estimates suggest that even a 1.5–2% reduction in milk yield due to mycotoxin exposure in dairy cows can lead to over $15,000 in annual losses for a 200-cow farm producing 8,500 liters per lactation (Additive, 2024). Moreover, the risk of mycotoxins, such as aflatoxin B (hepatotoxic and carcinogenic), roquefortine C (neurotoxic), citrinin (nephrotoxic), and ochratoxin A (both nephrotoxic and potentially carcinogenic), carries potential health hazards. This shows the challenge of managing fungal contamination in dairy products (Hymery et al., 2014). Aflatoxin M1 (AFM1), a metabolite of AFB1, is particularly concerning in dairy due to its heat stability. Chronic dietary exposure to AFM1 has been linked to hepatocellular carcinoma, especially in individuals co-infected with hepatitis B virus (Liu et al., 2012). The EU sets the maximum level for AFM1 in milk at 0.05 μg/kg, while the US FDA permits up to 0.5 μg/kg (FDA, 2019; EU, 2006). Additionally, Roquefortine C and citrinin, produced by certain *Penicillium* species in cheese, are known for their neurotoxic and nephrotoxic effects, although regulatory thresholds for these compounds remain less well defined (Finoli et al., 2001).

Fungal contamination in dairy products can occur at any stage from dairy farms to after reaching the consumer’s home, with sources ranging from unsanitary conditions to contaminated equipment and the addition of non-dairy ingredients (Garnier et al., 2017). The asexual spores (conidia) and vegetative cells of most mold species, along with yeasts, are sensitive to heat and typically do not survive pasteurization. However, they can cause food spoilage by producing heat resistant sexual ascospores and mycotoxins (Dagnas and Membré, 2013; Garnier et al., 2017; Pitt and Hocking, 2009). However, a small group of yeast species, *Debaryomyces, Saccharomyces*, and *Candida*, can survive heat processing and may cause spoilage of dairy products such as cheese and yogurt (Awasti and Anand, 2020). *Hamigara, Penicillium, Aspergillus,* and *Fusarium* are molds isolated from heat-treated dairy products, including cream cheese and pasteurized milk. In these molds, the heat-resistant sexual spores (ascospores) are responsible for their survival during pasteurization, or contamination may occur post-pasteurization (Garnier et al., 2017; Pitt and Hocking, 2009). Mold contamination in dairy factories is commonly linked to airborne transmission, as spores, mycelium fragments, and debris can readily spread through the air within these facilities. As demonstrated by Kure et al. (2004), air was identified as the primary carrier of significant cheese contaminants, such as *Penicillium commune* and *Penicillium palitans*, throughout the production process (Kure et al., 2004). To prevent airborne contamination, strategies such as high-efficiency particulate air (HEPA) filtration, positive air pressure systems, regular air quality monitoring, and strict sanitation protocols can be implemented to limit spore dispersion and accumulation in production areas (Garnier et al., 2017). Adding ingredients such as sweeteners, nuts, or fruits to dairy products, such as yogurt, can increase the risk of fungal spoilage by supplying additional sources of contamination and of nutrients that promote fungal growth and fermentation. Specifically, fruit additives, including blueberries and strawberries, are more susceptible to contamination because they cannot undergo extensive heat treatment and could contain fungi capable of forming heat-resistant spores (Penney et al., 2004). *Debaryomyces hansenii*, is difficult to control in fruit-flavored yogurt products, due to its high osmotolerance, resistance to low pH, and ability to grow at refrigeration temperatures. Its presence can cause defects in flavor, texture, odor, and color (Pilote-Fortin et al., 2021).

Notably, certain fungi contribute positively to dairy products. For example, *Penicillium camemberti* and *Penicillium roqueforti* are essential in ripening cheeses such as Camembert and Roquefort, contributing to their unique aroma and flavor (Chávez et al., 2012). Nevertheless, the proliferation of unwanted species can lead to spoilage or mycotoxin contamination (Kure and Skaar, 2019). LAB-based biopreservation strategies offer a targeted way to suppress spoilage fungi while maintaining the activity of beneficial mold cultures used in ripened cheeses (Shi and Maktabdar, 2022).

### Fungi in fruits and vegetables

Globally, between 20 and 30% of harvested fruits and vegetables are wasted each year, mainly due to decay caused by fungal contaminations occurring both pre-and post-harvest (Petrasch et al., 2019). More broadly, fungal plant pathogens are responsible for the destruction of up to 30% of total crop yield and contaminate approximately 25% of agricultural raw materials with spoilage fungi and mycotoxins (Nasrollahzadeh et al., 2022). These microorganisms predominantly follow necrotrophic or saprotrophic life cycles, catalyzing the decomposition of plant tissues either in the field, during harvesting, or post-harvest, ultimately resulting in the deterioration of the marketability of agricultural products. The production of mycotoxins by these fungi not only contaminates crops but also complicates food storage and preservation by requiring stringent measures to prevent further fungal growth and toxin accumulation (Bano et al., 2023).

Fungal infections in fruits involve four stages: spore adhesion, secure attachment, tissue invasion, and spread (Filippovich and Bachurina, 2022). In response, fruits activate antifungal defenses, which involve boosting phytohormone production, triggering an oxidative response, activating enzymes related to defense, and increasing the production of proteins that combat pathogens (Apaliya et al., 2017). Environmental parameters, both intrinsic (such as water availability, substrate composition, and pH) and external (such as humidity, temperature, and water activity), along with surrounding microorganisms, influence fungal infections and spore production (Bano et al., 2023). These factors collectively affect every stage of fungal growth, from spore germination to mycotoxin formation and mycelial development. Even with elevated water activity in vegetables and fruits, the relatively low pH, particularly in fruits, creates an environment favoring fungi over bacteria, leading to common mold spoilage (Bueno et al., 2007; Tournas, 2005).

The major fungi responsible for fruit and vegetable spoilage belong to the genera *Alternaria, Penicillium, Aspergillus,* and *Fusarium*. The mycotoxins they produce are Alternaria toxins (ATs), patulin (PAT), trichothecenes (TCs), and ochratoxin A (OTA). *Alternaria alternata* from the *Alternaria* genus causes mycotoxin contamination in a variety of crops, such as apples, strawberries, pears, melons, citrus, tomatoes, and potatoes (Logrieco et al., 2009). *Aspergillus* species, especially *A. flavus*, *Aspergillus niger*, and *Aspergillus ochraceus*, can infect plant tissues and produce certain types of mycotoxins, including fumonisins, aflatoxins, patulin, and ochratoxin A, which are found in seeds and fruits (Sanzani et al., 2016). *A. niger* is the predominant species causing decay in harvested fruits, such as citrus, apples, pears, peaches, grapes, figs, and strawberries. While spoilage in these fruits is often considered minor in terms of economic or health impact, the spoilage of crops such as tomatoes, onions, and garlic can lead to more significant postharvest losses under certain conditions (Plascencia-Jatomea et al., 2014). Most research focused on mycotoxins in fruits primarily centered around the toxin patulin, mainly synthesized by *Penicillium expansum* (derived from apples), and ochratoxin A, predominantly produced by *Aspergillus carbonarius* (derived from grapes and wines) (Zhang et al., 2017). Patulin contamination is a significant concern, considering that many apple-based processed foods are destined for infant nutrition (Sarubbi et al., 2016). Patulin exhibits genotoxic and immunotoxic effects. The EU and WHO set maximum permitted levels of patulin at 50 μg/kg for fruit juices and 10 μg/kg for baby foods (European Commission, 2003). OTA is frequently detected in grape-derived products and is considered a possible human carcinogen. *Aspergillus carbonarius*, prevalent in vineyards, is a major contributor to OTA accumulation during postharvest handling. The EU limit for OTA in grape juice and wine is 2 μg/kg (European Commission, 2006). Pathogens that cause decay in the storage phase often come from fields or orchards. The spores of mycotoxigenic isolates are found in the fruits of trees, yet they usually do not initiate growth or mycotoxin production until post-harvest (Sarrocco and Vannacci, 2018). Ochratoxin A is a globally recognized contaminant in grapes, wine, and other grape-derived products, such as juice or must. Black *Aspergilli*, especially *A. niger* and *A. carbonarius*, are common in vineyards-acting as primary contributors to the production of this toxin in grapes. During the postharvest phase, fungal species such as *A. carbonarius* become dominant, with *A. carbonarius* recognized as the most potent producer of ochratoxin A (OTA) in grapes (Sanzani et al., 2016).

Despite continuous efforts to eliminate mycotoxins, their presence in agricultural produce remains unavoidable. To reduce the risk of mycotoxin production, dissemination, and mold growth, it is necessary to develop and implement effective strategies that include three major components: Hazard Analysis Critical Control Points, Good Agricultural Practices, and emerging methods such as non-thermal preservation technologies and biological control approaches. These elements are key to minimizing the presence and impact of mycotoxins in food and feed (Paster and Barkai-Golan, 2008).

While fungal contamination in fruits and vegetables is typically associated with spoilage, certain fungi also serve protective roles. Species such as *Trichoderma harzianum* and *Aureobasidium pullulans* are used in postharvest biocontrol of fruits such as apples, grapes, and strawberries, where they suppress pathogens such as *Penicillium expansum* and *Botrytis cinerea* through competition, antibiosis, and mycoparasitism (Ippolito et al., 2000; Yao et al., 2023). In addition to fungal biocontrol agents, LAB-derived coatings and metabolites have shown promise in reducing surface spoilage fungi, thereby extending shelf life without affecting the fruit’s microbial balance (Ranjith et al., 2022).

### Fungi in meat products

Although the muscles of healthy animals are generally considered sterile, meat and meat products are susceptible to contamination during all stages of slaughter, preparation, and processing. Thus, the microbial ecosystem of meat and meat products is rich and diverse (Chaillou et al., 2015). The availability of nutrients, high water activity (nearly 0.99), and pH of 5.5 present favorable conditions for the propagation of a variety of microorganisms. Immediately after slaughter, most bacteria found on the carcasses are Gram-positive, of which 99% are mesophilic. With increasing storage time and low storage temperature, Gram-negative psychrotrophic bacteria gradually become dominant under aerobic conditions. When anaerobiosis sets in gradually, *Lactobacillus* and other facultative aerobic microorganisms, such as *Enterobacter* and *Brochothrix,* as well as fungi and mold, dominate (Hazards et al., 2023).

The fungi and molds proliferate in processed meat products such as fermented, dry-cured, or frozen meat (Mastanjević et al., 2023). Because of fermentation, ripening stages, and handling conditions, the physicochemical properties of these products become more suitable for contamination with a variety of beneficial and undesirable fungi and molds. Beneficial fungi improve desirable food properties by enhancing flavor quality through the secretion of specific enzymes such as lipases and proteases. Beneficial species such as *Penicillium nalgiovense* and *D. hansenii* contribute to flavor and surface protection, while undesirable fungi such as *Penicillium commune*, *A. flavus*, and *Mucor* spp. are associated with spoilage, off-odors, discoloration, or mycotoxin production (Hernandez-Mendoza et al., 2009). Others are toxigenic fungi and molds, which lead to undesirable odors and flavors, spoilage, and mycotoxin contamination. The growth of spoilage molds and yeasts in meat products is highly influenced by temperature and humidity. Most fungi, including *Penicillium* and *Cladosporium* species, thrive at temperatures between 15–30°C and relative humidity above 85%, particularly during the ripening and storage of dry-cured meats (Alves Rodrigues et al., 2025). Uncontrolled humidity and temperature in storage or processing facilities can accelerate spoilage and increase the risk of visible mold formation or mycotoxin production.

Fungal ecosystems of meat products are rich and diverse. A myriad of yeast species are obtained from meat products undergoing fermentation, such as *Rhodotorula mucilaginosa*, *D. hansenii*, *Cryptococcus* strains and *Candida* genus. It was also shown that this yeast population was dominated by *Yarrowia lipolytica*, *Candida zeylanoides* (a synonym of *Debaryomyces hansenii* according to some classifications) and basidiomycetous yeasts. While some of these yeasts, particularly *D. hansenii* and *C. zeylanoides*, play beneficial roles in flavor development, lipid breakdown, and surface stabilization during fermentation and ripening, others such as *Y. lipolytica* may contribute to spoilage under suboptimal storage conditions. In dry-cured Parma ham, Simoncini et al. (2007) identified a yeast population dominated by *D. hansenii*, *C. zeylanoides*, *Debaryomyces maramus*, *Hyphopichia burtonii,* many of which are considered technologically important for flavor and texture development in artisanal meat products (Simoncini et al., 2007). While meat is less commonly associated with dietary mycotoxin exposure, fungal metabolites such as OTA and citrinin can be introduced through contaminated spices or additives (Pleadin et al., 2021). OTA is a concern due to its stability and has been found in dry-cured meats (Toman et al., 2024). The EU limits OTA in meat products are not universally established, but stricter controls exist for spices (e.g., 15 μg/kg in nutmeg and paprika), which are often added to processed meats (Leprêtre and Merten-Lentz, 2018). Although financial estimates specific to fungal spoilage in meats are limited, case studies have shown OTA contamination rates of up to 100% in dry-cured hams from both industrial and household production sources (Pietri et al., 2011). Such contamination can lead to product rejection, recall, and reputational damage, underscoring the need for robust fungal control measures (Chen et al., 2022).

On the beneficial side, surface-ripened fermented meats often rely on intentional fungal colonization (such as *Penicillium nalgiovense*) to prevent oxidation and protect against spoilage organisms (Bernáldez et al., 2013). LAB strains used in dry-cured meats also contribute to microbial stability by producing organic acids and bacteriocins, which inhibit spoilage fungi while preserving the function of surface molds such as *Penicillium nalgiovense* (Laranjo et al., 2019).

### Food mycotoxins and their health effects

Mycotoxins are resistant to many microbiological food stabilization techniques, such as heating (Oliveira et al., 2015). As a result, humans and animals that ingest tainted food and feed are exposed to the toxic effects of these toxins. However, some mycotoxins possess significant antibiotic properties, which can exert selective pressure on microbial populations, promoting the emergence of resistant bacterial strains. This is particularly concerning in the gut microbiota of humans and animals consuming contaminated food, as it may reduce the efficacy of clinically important antibiotics such as penicillin and disrupt microbial balance (Modi et al., 2014). Mycotoxins can contaminate on many types of foods, such as fruits/dried fruits, nuts, spices, cereals, grains, and cheese at any point during the storing, harvesting, or production phase (Patel et al., 2021). Their occurrence is influenced by factors such as high humidity, elevated temperatures, insect damage, and poor storage conditions, which create favorable environments for fungal growth and toxin production.

**Figure 1:**
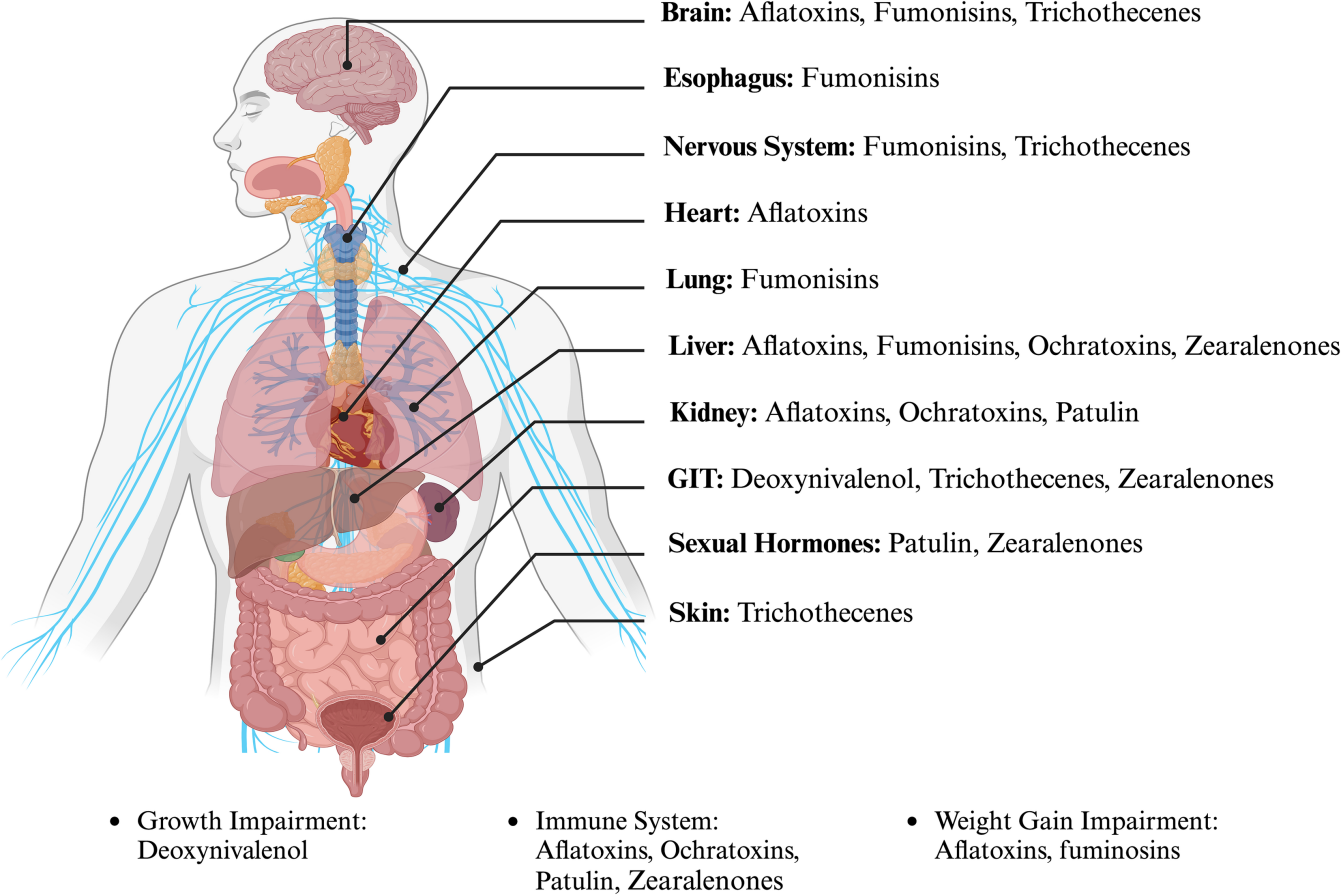
The most common foodborne mycotoxins and their impact on human organs. (Created with BioRender.com).

A few 100 mycotoxins have been identified, with approximately 30 of them found in mold-contaminated food and feed (Zhang et al., 2016). The main foodborne mycotoxins of public health concern are fumonisins, aflatoxins, trichothecenes, zearalenone, and ochratoxin A, although there are many others (Nguegwouo et al., 2018; Wu et al., 2014). Mycotoxins have been linked to mild and long-term human illnesses and can cause cancer in various organs, such as the liver, lungs, and kidneys, illustrating that harmful foodborne mycotoxins can affect human health (Figure 1).

Numerous studies have shown that foodborne mycotoxins can induce both acute and chronic toxicity, with carcinogenic, nephrotoxic, hepatotoxic, immunotoxic, teratogenic, and neurotoxic effects in humans and animals (Milićević et al., 2010). For instance, aflatoxin B1, produced by *A. flavus* and *A. parasiticus*, is one of the most potent naturally occurring liver carcinogens and has been classified by the International Agency for Research on Cancer (IARC) as a Group 1 carcinogen (Mohd Redzwan et al., 2016). Its carcinogenic effect is primarily due to the formation of DNA adducts, particularly AFB1–8,9-epoxide, which binds to guanine residues in DNA, leading to mutations in the TP53 tumor suppressor gene, a hallmark event in hepatocellular carcinoma (Bedard and Massey, 2006). Trichothecenes (e.g., T-2 toxin and deoxynivalenol) interfere with ribosomal function, inhibiting protein synthesis and triggering apoptosis, immunosuppression, and gastrointestinal distress (Hooft and Bureau, 2021).

Mycotoxins are prevalent in various foods, including some fermented and ripened products where molds may develop during processing or storage (Chelule et al., 2010). According to Marín et al. (2018), oilseeds, cereals, dried fruits, spices, flour, milk products, coffee, and other by-products are primary commodities that facilitate fungal growth and mycotoxin production. While there is overlap with the commodities listed in Table 1 (e.g., cereals, dried fruits, and spices), this list also includes additional categories such as oilseeds, flour, and milk products, suggesting that fungal contamination and mycotoxin risks extend across a broader range of food products (Marín et al., 2018). The most prevalent fungus in cheese is *Penicillium*, and *Penicillium expansum* produces patulin, a carcinogen that is more potent than the heterocyclic aromatic amines citrinin, polycyclic aromatic hydrocarbons, and nitrosamines. Fumonisins are produced mainly by *Fusarium* species, which can grow well on maize and foods made with maize is thought to cause swelling and throat cancer (Smith, 2018). Fumonisin disrupts sphingolipid metabolism by inhibiting ceramide synthase, a critical enzyme for maintaining cell membrane integrity and signaling. This mechanism has been associated with esophageal cancer and neural tube defects in high-exposure regions such as parts of China and South Africa (Voss et al., 2002). Zearalenone, another mycotoxin produced by *Fusarium* spp., mimics estrogen and binds to estrogen receptors, leading to reproductive disorders and potential endocrine disruption, in individuals and livestock (Sajjad et al., 2025). In grains, *Aspergillus* and *Penicillium* species are primarily responsible for producing ochratoxins. *A. parasiticus* and *A. flavus* are the two major producers of aflatoxin, and both prefer milk products as substrates (Makau et al., 2016). Ochratoxin A exhibits nephrotoxicity and has been linked to Balkan endemic nephropathy and renal tumors. The toxicity of OTA is associated with oxidative stress, inhibition of protein synthesis, and DNA damage, though its carcinogenic classification remains under IARC Group 2B (possible human carcinogen) (Bui-Klimke and Wu, 2015). Table 1 summarizes the most commonly encountered foodborne mycotoxins, the fungi that produce them, and the food products that harbor them.

**Table 1:** Common mycotoxins in food products and fungal species associated with their production.

Food source	Mycotoxins	Fungal species	References
Cereals	Aflatoxins	*A. flavus, A. parasiticus*	Abbès et al. (2016), Chen et al. (2018)
	Ochratoxins	*A. ochraceus, A. carbonarius, A. niger*	JØrgensen (2005), Szőke et al. (2022)
	Zearalenone	*F. graminearum, F. culmorum, F. cerealis*	Ghazvini et al. (2016), Shephard (2008)
	Trichothecenes	*F. graminearum, F. culmorum*	Beasley (1989), Woloshuk and Shim (2013)
Dairy and eggs	Aflatoxins	*A. flavus, A. parasiticus*	Embaby et al. (2022), Taniwaki et al. (2018)
Fruits and juices	Ochratoxins	*A. ochraceus, A. carbonarius, A. niger*	Serra Bonvehí (2004), Zimmerli and Dick (1996)
	Patulin	*Penic. expansum*	Nan et al. (2022), Wright (2015)
Meat	Aflatoxins	*A. flavus, A. parasiticus*	Abbès et al. (2016), Chen et al. (2018)
Nuts and dried fruits	Aflatoxins	*A. flavus, A. parasiticus*	Embaby et al. (2022), Taniwaki et al. (2018)
Vegetables	Aflatoxins	*A. flavus, A. parasiticus*	Chen et al. (2018), Embaby et al. (2022)
Maize and processed products	Fumonisins	*Fusarium verticillioides, F. proliferatum*	Blanchard and Manderville (2016), JØrgensen (2005)
Wine and coffee	Ochratoxins	*A. ochraceus, A. carbonarius, A. niger*	Blanchard and Manderville (2016), JØrgensen (2005)
Spices and beans	Ochratoxins	*A. ochraceus, A. carbonarius, A. niger*	Serra Bonvehí (2004), Szőke et al. (2022)

Penic, Penicillium.

### Preventing mold growth: LAB as a valuable source of antimicrobial compounds

**Figure 2:**
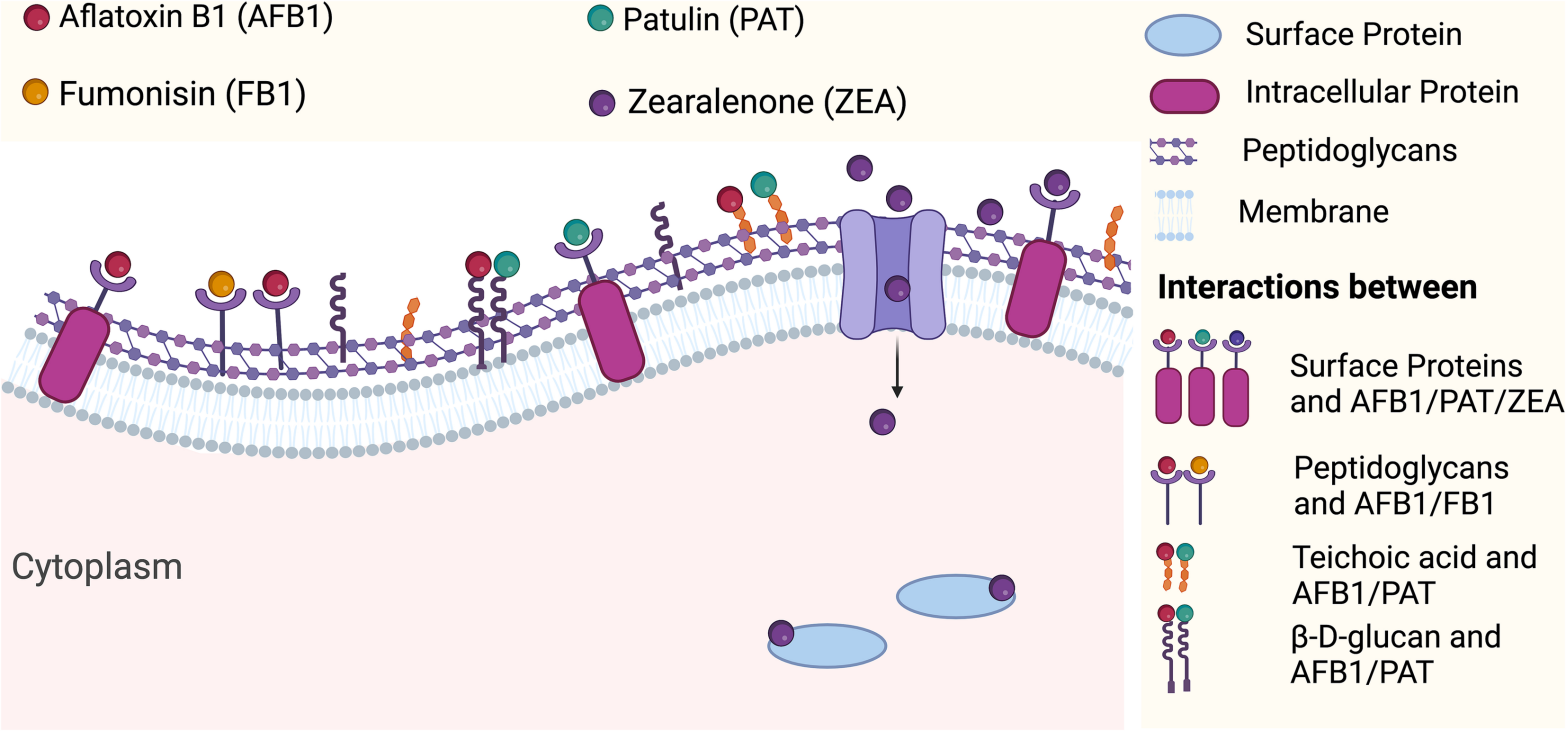
Antimicrobial compounds from LAB strains and their inhibitory effects on spoilage microbes. (Created with Biorender.com).

LAB have antimicrobial capabilities that hinder the proliferation of fungi and various Gram-positive and Gram-negative bacteria, justifying their application in food fermentation, preservation, and storage. LAB can produce a range of antimicrobial substances including organic acids, reuterin, hydrogen peroxide, hydroxylated fatty acids, exopolysaccharides, and bacteriocins. These compounds exhibit distinct modes of action that contribute to microbial inhibition and shelf life extension in food systems (Figure 2).

Among the low molecular weight metabolites, organic acids such as lactic, acetic, and propionic acids acidify the food matrix and reduce pH, creating unfavorable conditions for fungal growth. Diacetyl interferes with microbial metabolism by reacting with amino groups, while hydrogen peroxide induces oxidative stress via reactive oxygen species that damage fungal membranes and DNA (Liu A. et al., 2022). Reuterin, produced by *Limosilactobacillus reuteri*, is a broad-spectrum antimicrobial that functions by alkylating thiol groups in proteins and enzymes of spoilage organisms. While its synergy with organic acids such as lactic acid has been demonstrated against bacteria, whether this interaction enhances antifungal activity remains to be confirmed (Soltani et al., 2022).

Some LAB strains, notably *Lactiplantibacillus plantarum,* can hydroxylate unsaturated fatty acids to produce hydroxy derivatives such as 3-hydroxytetradecanoic acid and 3-hydroxydodecanoic. These compounds exhibit antifungal properties by integrating into fungal membranes and disrupting their integrity (Sjögren et al., 2003). These molecules are particularly effective against food spoilage yeasts and molds in low-pH environments typical of fermented foods (Nasrollahzadeh et al., 2022).

Extracellular polymeric substances (EPS), produced by certain LAB strains, are multifunctional secondary metabolites. While primarily known for improving food texture and mouthfeel (biothickening), some EPS also exhibit antiadhesive or biofilm-inhibitory properties, reducing the ability of fungi to colonize surfaces in food systems (Kavitake et al., 2023). In addition, EPS may indirectly contribute to antifungal activity by promoting LAB competitiveness and persistence in food environments. EPS from LAB are also known to stimulate immune responses and modulate gut microbiota, offering dual functionality in health and preservation (Jurášková et al., 2022).

Bacteriocins, another important group of LAB metabolites, are ribosomally synthesized proteinaceous molecules produced by LAB that primarily target bacteria. Although they are predominantly known for their antibacterial activity, certain strain-specific bacteriocin-like inhibitory substances (BLIS), such as those produced by *Pediococcus pentosaceus*, have been reported to exhibit antifungal or antimycotoxigenic effects in specific contexts, particularly in silage and feed matrices (Dalié et al., 2010; Souza De Azevedo et al., 2020). However, the mechanisms responsible for these effects remain insufficiently studied, and such activity is not consistently observed across LAB strains or food systems. Nisin, produced by *Lactococcus lactis*, is the most extensively studied and the only bacteriocin currently approved for use in food. It is widely applied in dairy and meat products to control spoilage and pathogenic bacteria. Nisin acts by binding to lipid II, a precursor in bacterial cell wall synthesis, leading to pore formation and cell lysis (Field et al., 2023). While primarily active against Gram-positive bacteria, some studies suggest nisin may also inhibit mold spore germination or indirectly reduce fungal colonization by suppressing associated bacteria that facilitate fungal growth (Gut et al., 2008). Bacteriocins are considered promising biopreservatives due to their neutral sensory profile and stability across a broad range of pH, temperature, and salt conditions. They can be delivered as bioactive powders or incorporated into antimicrobial packaging, offering extended protection without altering food quality. Limitations such as narrow activity spectrum or inactivation by food components may be addressed through encapsulation techniques or combined preservation strategies (Bastos et al., 2015).

### Antifungal compounds from LAB

The family Lactobacillaceae is one of the dominant groups in the food microbiome and is strongly associated with antifungal activity. Extending shelf life while maintaining stability and safety in foods is the primary objective, typically achieved by inhibiting pathogenic microorganisms and spoilage. Various antimicrobial agents may be used alone or synergistically to inhibit the proliferation of spoilage microorganisms and foodborne pathogens. Importantly, these agents preserve the nutritional and sensory characteristics of foods, maintaining their physicochemical structure. In addition to their antifungal properties, LAB are beneficial in food products as they can: (1) limit the proliferation of hazardous enteric pathogens, (2) provide beneficial enzymes, (3) eliminate toxic food components in the gut, (4) enhance the immune system, and (5) stimulate peristaltic movement of food through the gastrointestinal tract. Table 2 summarizes the LAB strains obtained from various sources, along with their antifungal activity spectra. Membrane instability, enzyme inhibition, the formation of oxygen-reactive species, and proton gradient interference (Schaefer et al., 2010) are some of the mechanisms behind the inhibition of single compounds that have been explored in depth (Figure 2). However, many investigations have focused solely on the effects of individual compounds, ignoring the synergistic and additive effects of compounds used in combination. Future studies should explore optimized combinations of LAB-derived metabolites, their mechanisms of interaction, and their efficacy in complex food matrices under real storage conditions.

**Table 2:** Antifungal activity spectrum of lactic acid bacteria sourced from a diversity of food and feed.

Strain	Source	Targets	Action	Active compound	References
*Lcb. casei; L. delbrueckii* spp.	Dairy	*Penicillium* spp. *Diutina rugosa*	Proteolytic activity; Unknown	CFS	Erginkaya et al. (2004), Girardin (1997)
*L. helveticus*		*Penicillium* sp.	Proton gradient interference	LA, AA	Bian et al. (2016)
*Lcb. rhamnosus MDC 9661; Sch. harbinensis K. V9.3.1 Np*		*Various fungi, including Penic. thomii*	Linked with cell wall: Yeast membrane depolarization	LBC; LA, CFS	Bazukyan et al. (2018), Mieszkin et al. (2017)
*Liml. fermentum* (Cassava, Andean products)	Fermented Products (Non-Dairy)	*Aspergillus* spp.*, Penic. expansum*	Unknown; Proton gradient interference	CFS; PLA, 3,5-Di-O-caffeoylquinic acids	Adedokun et al. (2016), Yépez et al. (2017)
*Liml. fermentum C14 (Curd)*		*Penic. digitatum, Mucor* sp.	Proton gradient interference	LA, PLA, CFS	Barman et al. (2017)
*Fruct. sanfranciscensis CB1; Lcb. paracasei* subsp. *tolerans*	Sourdough	*Various fungi including Fusarium, Penicillium*	Unknown	Mixture of organic acids	Corsetti et al. (1998), Hassan and Bullerman (2008)
*Furfl. rossiae; Liml. reuteri CRL 1100, Levil. brevis CRL 772/796*		*A. niger, Penic. roqueforti; Aspergillus, Fusarium*	Proton gradient interference	LA, AA, PLA	Gerez et al. (2009), Valerio et al. (2009)
*Secund. paracollinoides*	Vegetable	*F. graminearum, Botrytis cinerea*	Unknown	LBC	Sathe et al. (2007)
*Liquor. mali VLT112, Lcp. pentosus VLT310 (Salami)*	Meat	*A. candidus, Penic. nalgiovense*	Proton gradient interference, enzyme inhibition	PLA, HO-PLA	Corsetti and Settanni (2007)
*Luig. coryniformis Si3*	Grass silage	Various fungi including *F. sporotrichoides*	Unknown	Partially purified compounds	Magnusson and Schnürer (2001)
*Liml. fermentum*	Cocoa bean	*Penic. citrinum, G. moniliformis*	Proton gradient interference	AA, LA	Romanens et al. (2019)

AA, Acetic acid; LA, Lactic acid; BA, Butyric acid; PA, propionic acid; FA, Formic acid; PLA, Phenyllactic acid; SA, Salicylic acid; LBC, Live bacterial cell; CFS, Cell free supernatant; PCO, Proteinous compound; UN, Unknow; Penic, Penicillium.

### Mycotoxin detoxification by LAB species

Mycotoxin detoxification by LAB species involves detoxification of various mycotoxins. Biological detoxification uses microorganisms and metabolites to biotransform mycotoxins into less harmful or harmless chemicals (Liu L. et al., 2022). Biological detoxification is a promising option because hundreds of microorganisms and metabolites are available. The microorganisms used for detoxification must be safe, and capable of producing stable, nontoxic metabolites that degrade mycotoxins into harmless byproducts through irreversible chemical reactions. Additionally, they must avoid generating undesirable odors or tastes, possess minimal cultivation and production requirements, remain active throughout storage, and preserve the nutritional value of the food. Several microbes have been proposed as food and feed detoxifiers, but only a few have been tested for practical use. Yeast, bacteria, and fungi are the microorganisms most commonly employed to detoxify feed and food (Shekhar et al., 2025). Karlovsky (1999) explored the prospect of using microbes such as *Rhizopus* sp., *A. niger*, *Yarrowia lipolytica* (formerly *Candida lipolytica*), *Mucor ambiguus, Neurospora* spp., *Trichoderma viride, Desarmillaria tabescens* (formerly *Armillariella tabescens*), and LAB for detoxification (Karlovsky, 1999). Owing to their established safety, LAB are the preferred microorganisms for degrading mycotoxins. LAB are chosen over other microbes because they are recognized as safe, occur naturally in the human digestive tract, and remain easy to cultivate and maintain. Lactic acid bacteria follow two pathways for food mycotoxin detoxification: (1) using the viable cells of microorganisms and (2) enzymes produced by specific LAB strains. Lactic acid bacteria proteolytic enzymes play important roles in detoxifying mycotoxins in food (Nichea et al., 2015; Wu et al., 2009). The use of LAB to reduce mycotoxins in food has been investigated extensively (Table 3). Integrating LAB cells and their metabolites to reduce mycotoxin levels in food offers several benefits. The adsorption of mycotoxins by the LAB cell wall has been proposed as a potential strategy for mycotoxin removal from food. This action involves polysaccharides, proteins, and cell walls of LAB strains that contain peptidoglycans (Chapot-Chartier and Kulakauskas, 2014). The binding activity of specific LAB strains has been shown to decrease patulin levels in culture media (Wang et al., 2015). This study suggests that LAB strains with thicker cell walls and greater surface areas can improve their binding to and neutralization of patulin, leading to mycotoxins exclusion. Polysaccharides and proteins have been reported to be critical functional components for the adsorption of patulin. Another study concluded that mycotoxin binding by LAB cells was dependent on the initial mycotoxin concentration, LAB cell count, food complexity, pH, and incubation temperature (Luz et al., 2018). Research conducted by Dalié et al. (2010) showed that living bacterial cells in yogurt are not essential for the detoxification of aflatoxin B1. This is because aflatoxin B1 binds to specific components on the cell wall from dead bacteria, such as polysaccharides or proteins, facilitating its neutralization in yogurt (Dalié et al., 2010).

**Table 3:** Mycotoxin detoxification by LAB species or strains.

Target mycotoxin	Microorganism (species or strain)	Degradation (%)	Culture form	References
Aflatoxin (AFB1, AFB2, AFG1, AFG2)	*L. delbrueckii*	>99%	LBC	Saladino et al. (2016)
AFB1	*L. amylovorus; Levil. brevis LOCK 0944 (Note: This appears twice in the list) Lent. buchneri; L. bulgaricus; Lcb. casei; Lcb. casei LOCK 0920; Lcb. paracasei LOCK 0920; Lpb. plantarum; Lpb. plantarum C88; Lpb. plantarum LOCK 0945 (Note: This appears twice in the list); Lpb. plantarum MYS44; Lpb. plantarum Lcb. rhamnosus; Lcb. rhamnosus GG; Lcb. rhamnosus LC-705; Lpb. plantarum Lactococcus lactis Pedioc. acidilactici; S. thermophilus*	11–81%	LBC/CFS	Poornachandra Rao et al. (2019), Sezer et al. (2013)
Ochratoxin (OTA)	*B. animalis* subsp*. lactis VM12; L. acidophilus VM 20; Levil. brevis LOCK 0944; Lent. buchneri; Lcb. casei LOCK 0920; Liml. fermentum; Lpb. plantarum; Lpb. plantarum LOCK 0945; Liml reuteri; Lcb. rhamnosus CECT 278 T; Pedioc. parvulus*	50–97%	LBC	Fuchs et al. (2008), Luz et al. (2018), Niderkorn et al. (2009)
Patulin	*Lent. buchneri;, Liml reuteri; Lpb. plantarum; Liml. fermentum; Enterococcus faecium; Lcb rhamnosus; L. acidophilus; Lpb. plantarum*	65–90%	LBC/HKBC	Hatab et al. (2012), Zoghi et al. (2017)
Zearalenone (ZEA)	*Pedioc. pentosaceus KTU05-9; Pedioc. acidilactici; Lcb. paracasei, L. lactis*	37–55%	LBC	Juodeikiene et al. (2018), Rogowska et al. (2019)

LBC, Live bacterial cell; CFS, Cell-free supernatant; HKBC, heat-killed bacterial cell; Pedioc, Pediococcus.

**Figure 3:**
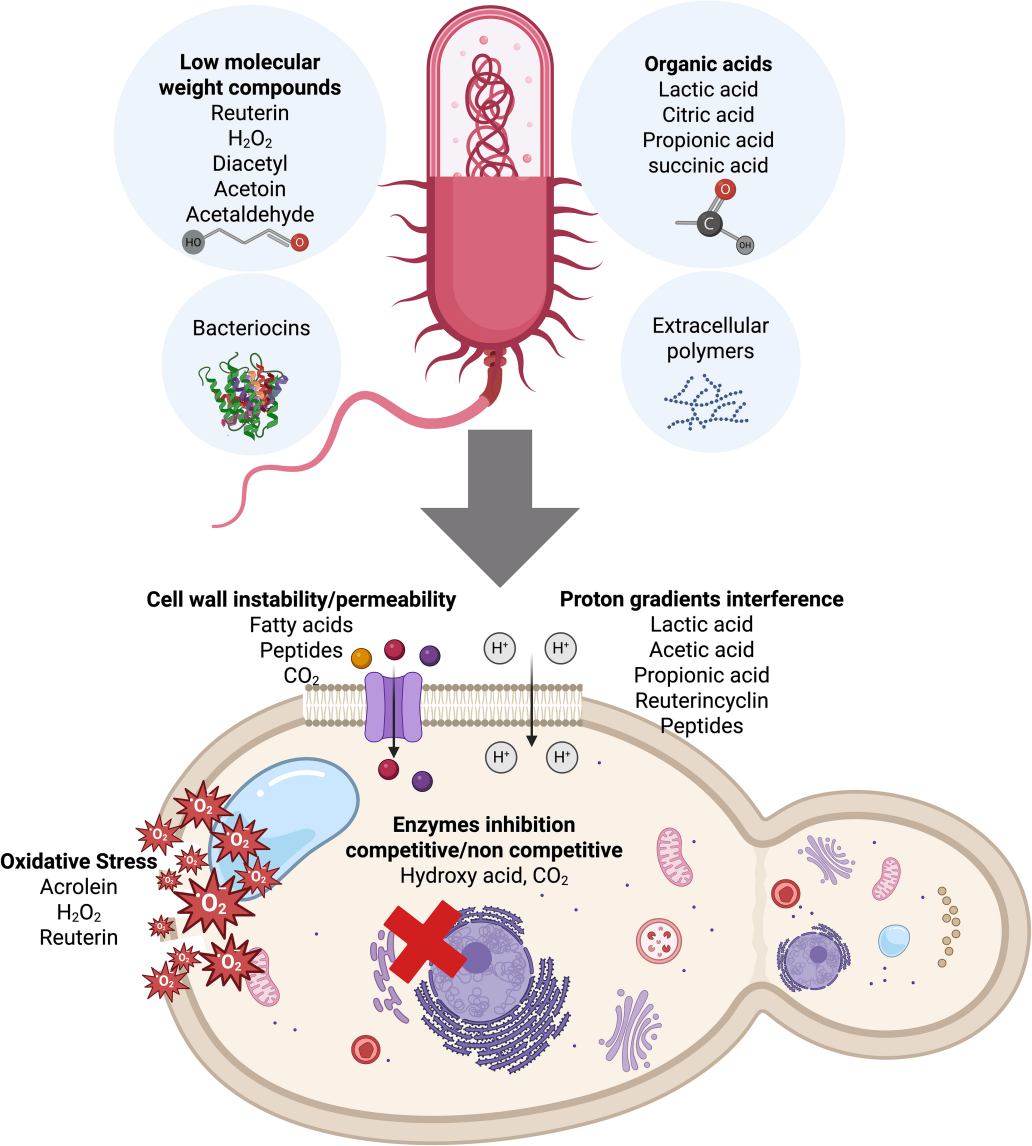
Mycotoxin interaction with bacterial cell walls. (Created with BioRender.com).

Metabolites produced by LAB strains, such as acids, low-molecular-weight bioactive peptides, phenolic compounds, and fatty acids, interact to reduce the levels of mycotoxins in foods (Niderkorn et al., 2009). These metabolites contribute to detoxification through diverse mechanisms, such as enzymatic degradation and chemical interactions with mycotoxins. The breakdown of mycotoxins and their elimination by metabolites and LAB cells are not yet fully understood (Muhialdin et al., 2020). However, hypotheses regarding various mechanisms have been proposed, including proteolytic enzyme activity and specific interactions between metabolites and mycotoxins. These interactions often lead to binding, sequestration, and in some cases, degradation of mycotoxins. For example, proteolytic enzymes may hydrolyze mycotoxin structures, while specific cell wall components, such as peptidoglycans, surface proteins, and teichoic acids, facilitate binding and potentially enhance enzymatic degradation (Liu L. et al., 2022) (Figure 3). Studies have demonstrated that LAB enzymes, cells, and metabolites can work simultaneously to degrade or neutralize mycotoxins, reducing their toxicity and prevalence in food systems (Muhialdin et al., 2020).

Lahtinen’s research group discovered that the binding of AFB1 by *Lacticaseibacillus rhamnosus* strain GG, is linked to cell wall peptidoglycans, emphasizing the importance of bacterial cell wall components in mycotoxin adsorption (Lahtinen et al., 2004). AFB1 binding is not associated with exopolysaccharides, minerals (calcium or magnesium), proteins, or lipids (Lahtinen et al., 2004). Similarly, teichoic acids from *Lacticaseibacillus casei* Shirota and *Liml. reuteri* NRRL14171 have been found to bind AFB1 (Hernandez-Mendoza et al., 2009). The binding ability of *Lcb. casei* Shirota cells to AFB1 is attributed to proteins on the bacterial cell wall. AFB1 may also attach to the bacterial cell wall D-glucans through van der Waals interactions and the formation of hydrogen bonds (Yiannikouris et al., 2006).

While binding interactions are crucial for sequestering AFB1, they may also facilitate degradation by creating proximity to enzymatic or chemical processes that break down the toxin. Environmental factors, such as pH and temperature, influence the cell wall structure and physicochemical properties, including electrostatic interactions, thereby affecting the availability of mycotoxin binding sites (Muhialdin et al., 2020). Acidic conditions, for instance, enhances hydrophobic interactions by breaking down surface proteins and exposing additional AFB1 binding sites. These interactions not only favor adsorption but may also enhance degradation pathways, underscoring the importance of understanding how structural and environmental factors contribute to improving LAB efficiency in reducing mycotoxin toxicity (Bueno et al., 2007).

#### Patulin

Patulin is a water-soluble mycotoxin produced by numerous *Aspergillus* and *Penicillium* species. However, the apple rot fungus *Penicillium expansum* is the most prevalent species involved in patulin synthesis (Plascencia-Jatomea et al., 2014). High levels are found in foods, such as grapes and grains. Patulin residues are problematic because they can enter tissues, stop protein synthesis, and lower glycogen levels in the intestines, kidneys, and liver. The critical binding structures of patulin are cell surface adhesion proteins. Recent studies have identified various parameters associated with patulin biosorption by inactivated LAB strains. According to Wang et al. (2015), pretreatment with esterification and NaOH improved patulin binding. In contrast, pretreatment with iodate, trypsin, periodate, and lipase reduced binding (Wang et al., 2015). The significance of vicinal and carboxyl OH groups was negated, with esters, alkaline amino acids, and thiol suggested as probable molecules implicated in adsorption. LAB strains adsorb patulin through hydrophobic and electrostatic interactions. Wang et al. hypothesized that greater patulin adsorption could be attributed to a larger cell area and volume, which varies among species and cell types based on differences in cell wall composition and structural characteristics. LAB carbohydrate components (NH, C-O, and OH groups) and cell surface proteins have also been identified as key factors influencing patulin adsorption (Wang et al., 2015). The information provided did not reveal the specific processes or interactions linking the bacterial cell wall attributes and mycotoxins (Figure 3). However, certain compounds, such as fructooligosaccharides and ascorbic acid, have been shown to enhance the patulin-binding capacity of *Lpb. plantarum* ATCC 8014 and *Lactobacillus acidophilus* ATCC 4356 by possibly altering the cell wall structure or increasing the availability of functional binding sites. This effect appears to be strain-dependent and may not be common across all strains within these species (Zoghi et al., 2017).

#### Fumonisins (FB)

Fumonisin is a mycotoxin primarily produced by *Fusarium proliferatum* and *Fusarium verticillioides* (formerly *F. moniliforme*). Among the fumonisins, B1, B2, and B3 are the most prevalent and toxic, with B1 being the most frequently detected and associated with significant health risks, such as carcinogenicity and disruption of sphingolipid metabolism (Anumudu et al., 2024). Fusarium-infected corn may sicken animals and induce equine leukoencephalomalacia (ELEM), a long-standing disease in North America. Fumonisins are found in corn, tortillas, sorghum, rice, and medicinal plants. Niderkorn found that fumonisin (FB1 and FB2) probably binds to peptidoglycan or molecules very similar to it without the help of surface lipids, proteins, or polysaccharides (Niderkorn et al., 2009) (Figure 3). The peptidoglycans of *Lpb. plantarum* B7 and *Lactiplantibacillus pentosus* X8 had maximum capacity to bind fumonisins compared to other LAB strains tested in the study, demonstrating their superior binding efficiency relative to the other strains evaluated (Zhao et al., 2016). The mechanism of fumonisin-LAB cell wall peptidoglycan interaction is unknown. The interaction of fumonisin tricarballylic acid chains occurs with peptidoglycans during binding (Niderkorn et al., 2009).

#### Zearalenone (ZEA)

Several *Fusarium* species produce zearalenone, a nonsteroidal estrogenic mycotoxin. *Fusarium graminearum* is the primary producer of ZEA. Some *Fusarium* species that produce zearalenone include *Fusarium sporotrichioides, Fusarium semitectum, Fusarium equiseti, Fusarium oxysporum, Fusarium culmorum,* and *F. verticillioides.* Contamination of cereal grains with zearalenone has been reported in warm climates (Milani, 2013). Generally, zearalenone concentrations are low in contaminated field grains, but tend to increase during storage, with moisture levels of 30–40% (Agriopoulou et al., 2020b). Zearalenone significantly affects female reproduction (hyperestrogenism) and male reproduction (Yang et al., 2007). Recent research indicates zearalenone poses serious human health risks (Belhassen et al., 2014; Mally et al., 2016). ZEA removal by LAB has been shown to be strain-dependent and influenced by factors such as the protein and lipid composition of the bacterial cell wall, bacterial concentration, and the presence of co-occurring mycotoxins, which may compete for binding sites. Heat and acid treatments have also been shown to significantly enhance ZEA and *α*-ZOL removal, while polysaccharides and pH appear to have limited effect (Adunphatcharaphon et al., 2021). From the initial adsorption stage of 720 min, this rate fell from 5.49 g/mL min^−1^ to 0.15 g/mL min^−1^ during the secondary adsorption, reaching equilibrium. LAB may remove ZEA through cell surface proteins, peptidoglycans, or absorption into bacterial cell interactions (Figure 3). Likewise, cell wall components of *Lpb. plantarum* strain 102 bind the T-2 toxin (Król et al., 2018). Trichothecene mycotoxin elimination by LAB strains is only known to occur through cell wall binding.

### Antifungal activity of LAB in bakery products

The use of LAB as preservatives is extensive, as reviewed by (Crowley et al., 2013; Shi and Maktabdar, 2022; Zapaśnik et al., 2022). It has been demonstrated that incorporating LAB into sourdough fermentation is an effective strategy to prevent mold spoilage in bread, as LAB can produce antifungal metabolites during the fermentation process. This effect is partially due to the generation of organic acids such as acetic or lactic acid (Le Lay et al., 2016). Additionally, it has been shown that acidifying dough significantly influences the qualitative features of bread, such as volume and texture (Jekle and Becker, 2012). Among the various LAB strains used in sourdough, *Levilactobacillus brevis* and *Lpb. plantarum* have been shown to have favorable effects on bread characteristics (Le Lay et al., 2016). Although the dominance of LAB species in sourdough has been shown, Ventimiglia et al. (2015) concluded that *Lpb. plantarum* usually manifests as co-dominant with heterofermentative LAB. Another species, *Lcb. casei*, is found in the microbiota of sourdough baked goods and has been employed in sourdough medium in several studies (García-Mantrana et al., 2016; Kitahara et al., 2005) Additionally, some studies have shown that this species can produce exopolysaccharides and has been verified as part of sourdough starter culture (Galle et al., 2011).

According to Ajith and Sunita (2017) LAB-treated bread did not show any fungal contamination for up to four days. This observation aligns with the shorter shelf life reported for some LAB strains, such as *Lactobacillus amylovorus* and *Liml. reuteri*, as indicated in Table 4, depending on the strain and specific application (Ajith and Sunita, 2017). The primary metabolites of LAB fermentation, including acetic/lactic acid, also hinder the proliferation of *Rhizopus* sp. and *Mucor* sp. to 40 and 20%, respectively. Di Biase et al. (2014) and Schieber and Saldaña (2009) observed *A. niger* growth after seven days of baking was slower in LAB-inoculated bread than in control bread. Axel et al. (2015) tested *L. amylovorus* DSM 19280 as a sourdough starter culture for antifungal activity (Axel et al., 2015). Instead of exhibiting mold after 2 days with control samples, it increased the shelf life of bread by an additional four days. In another study, Cizeikiene et al. (2013) tested *Pediococcus acidilactici* strains KTU05-10, KTU05-7, and KTU05-8 in sourdough. Adding sourdough generated with these strains to bread reduced fungal degradation and development throughout the storage period of 8 days, whereas the control group showed conspicuous fungal colonies (Cizeikiene et al., 2013). Additionally, *Lpb. plantarum* LB1 and *Furfurilactobacillus rossiae* LB5 were added to *Penicillium roqueforti* DPPMAF1 to determine how well they killed the fungi. After 21 days from inoculation, the wheat germ bread sample showed mycelial growth with only 10% contamination (Bullerman and Bianchini, 2007).

**Table 4:** Studies showing the use of LAB species and strains to improve the shelf life of bread.

Starter culture or strain	Shelf-life extension	References
*Lpb. plantarum* 1A7	Up to 28 days	Coda et al. (2011)
*Lpb. plantarum*	>14 days	Coda et al. (2011), Gardner et al. (2002), Gerez et al. (2015), Mo and Sung (2014)
*Pedioc. acidilactici* KTU05-7, *Pedioc. pentosaceus* KTU05-8, KTU05-10	8 days	Cizeikiene et al. (2013)
*Propionibacterium freudenreichii, Lpb. plantarum*	7 days	Ran et al. (2022)
*Lpb. plantarum*	4 days	Axel et al. (2015), Cizeikiene et al. (2013); Sadeghi et al. (2019)
*Levil. hammesii*	6 days	Black et al. (2013), Maldonado et al. (2015), Schieber and Saldaña (2009), Wei et al. (2009)
*L. amylovorus*	4 days	Axel et al. (2015)
*Liml reuteri, Levil. brevis*	2 days	Axel et al. (2015), Cizeikiene et al. (2013), Ribes et al. (2018), Zhang et al. (2010)

Pedioc, Pediococcus.

*Lentilactobacillus diolivorans* and *Lentilactobacillus buchneri*, two active propionate producers, were applied to mold-damaged bread to inhibit fungal growth and improve shelf life. Zhang et al. (2010) demonstrated that these strains effectively suppressed mold growth for over 12 days (Zhang et al., 2010). In addition, Ran et al. (2022) found that a mixed culture of *Propionibacterium freudenreichii* D6 and *Lpb. plantarum* L9 significantly delayed the growth of *A. niger* in bakery systems, with acetic acid being the primary contributor to antifungal activity (Ran et al., 2022). Interestingly, *in situ* spraying of bakery products with selected strains of *Liml. reuteri* 5,529, *Levilactobacillus spicheri* O15, and *Leuconostoc citreum* L123 delayed fungal growth when sprayed directly onto pound cake and milk bread rolls contaminated with spoilage fungi, showing strong in situ antifungal activity.

A recent study identified three new LAB strains (*Lpb. plantarum* jQ 301,799, *Lcb. casei* jQ 412,732, and *Levil. brevis* IBRC-M10790) isolated from Tarhana (Tafvizi and Tajabadi Ebrahimi, 2015) in sourdough. The study evaluated the bacterial attributes of these strains and their impact on the texture and preservation of toast over a six-day storage period, showing the production of diverse metabolites by LAB. Additionally, this study aimed to evaluate the potential synergistic or antagonistic effects of LAB combined in sourdough. Settanni et al. (2011) identified *Lpb. plantarum* and *Levil. brevis* among the predominant LAB strains during tarhana fermentation, showing how controlled fermentation parameters influence microbial diversity and support their potential use in bio-preservation (Settanni et al., 2011). Hadaegh et al. (2017) explored the effect of sourdough infused with three newly identified individual LAB strains (*Levil. brevis* IBRC-M10790, *Lpb. plantarum* jQ 301,799, and *Lcb casei* jQ 412,732), and mixed strains on the qualitative attributes of toast bread. The study assessed parameters such as microbial preservation, texture, and bread staling. While the sourdough concentration significantly enhanced microbial preservation by inhibiting spoilage microorganisms, it had minimal effect on the decrease in enthalpy, which reflects the thermal stability or structural integrity of the bread during storage. Mixed LAB strains produced the highest quantity of organic acids, lowering the enthalpy and hardness of bread and improving microbial preservation. Among single strains, *Lcb. casei* reduced bread hardness, improved bread volume, and had the best staling rate (Hadaegh et al., 2017).

### LAB antifungal potential in dairy foods

The antifungal activity of LAB has been demonstrated in a number of fermented dairy products, such as cheese and yogurt (Garnier et al., 2017). In addition, LAB strains from species such as *Lpb. plantarum*, *Lcb casei,* and *Lcb. rhamnosus* have shown antifungal effects, which could result in extended shelf-life of dairy products (Leyva Salas et al., 2017). Souza et al. (2023) showed that various LAB strains isolated from cheese and dairy settings hinder the growth of *A. niger* IOC 207 and *Penicillium chrysogenum* IOC 132 (Souza et al., 2023). This suggests that they may be used as biocontrol agents to expand the shelf life of cheese as an alternative to chemical preservatives or thermal processing. Leyva Salas et al. (2018) conducted a study testing two combinations of LAB strains, A1 and A3, for their antifungal effects on dairy products such as sour cream and semi-hard cheese. Both combinations included *Lpb. plantarum* L244, paired with either *Schleiferilactobacillus harbinensis* L172 (A1) or *Lcb. rhamnosus* CIRM-BIA1113 (A3). The A1 combination notably delayed fungal growth in sour cream for up to 24 days and in semi-hard cheese for up to 6 days (Leyva Salas et al., 2018). In addition, Cosentino et al. (2018) concluded that a combination of four Lactobacilli strains could control mold on caciotta cheese without affecting the taste, suggesting the potential of LABs in prolonging the shelf life of dairy (Cosentino et al., 2018).

The antifungal properties of LAB are linked to their ability to generate metabolites, such as fatty acids, organic acids, and proteins. Notably, *in situ* production of these antifungal metabolites by LAB cultures yielded concentrations significantly lower than the minimum inhibitory concentrations (MICs). Importantly, this means that LAB metabolites may interact synergistically with each other (Cosentino et al., 2018). The ability of LAB to deplete manganese (Mn) presents a novel strategy for slowing the proliferation of fungal spoilage in dairy foods, offering the dairy industry a non-destructive means of controlling fungal contamination. Supporting this mechanism, *Lcb. rhamnosus* LRH01 and *Lpb. plantarum* LP01 have been shown to inhibit the growth of *Penicillium* strains commonly found in dairy products, both in laboratory media and yogurt serum. Notably, when Mn was reintroduced into the yogurt matrix, the antifungal effect was reduced or completely lost, indicating that manganese depletion is essential for inhibiting *Penicillium* growth (Shi and Maktabdar, 2022). Predicting mold sensitivity and the success of bioprotective cultures in food preservation requires consideration of multiple factors, including the composition of the food matrix, such as Mn levels, and storage conditions (Shi and Maktabdar, 2022). Understanding the complex interactions between LAB and spoilage fungi across various environments is required to optimize inhibitory strategies.

### Antifungal activity of LAB in fruit and vegetable products

LAB are key contributors to the protection of fruits and vegetables against fungal attack and mycotoxin contamination. Chen et al. (2021) isolated 13 strains with fungicidal activity out of 224 LAB strains from cured pickles. Among the tested strains, *Lpb. plantarum* CWXP24 and *Lpb. plantarum* CKXP13 showed the highest efficacy against *Penicillium digitatum* on citrus fruits, inhibiting decay and reducing lesions (Chen et al., 2021). *Lcb. casei* YZU01 broke down patulin in fresh apple and pear juices within 36 h of incubation, showing promise for toxin removal in commercial juice products.

*Leuconostoc mesenteroides* subsp. *mesenteroides* (LB7), grown from apple skin, has not only antifungal activity but also reduces the levels of patulin, although with variable efficiency depending on the type and juice contamination level. The factors determining the efficacy of LAB include pH (5.5–7.0 for optimal antifungal production), bacterial concentration, nutrient availability (glucose at 1.5% enhances production in certain media), competitive bacteria, and incubation conditions (temperature and duration). These conditions not only influence the growth of LAB but also the yield and activity of their antifungal substances (Dalié et al., 2010).

LAB strains are used not only for reducing microbial contamination on produce but also for extending its shelf life. While applications of LAB are currently under consideration for food systems, close scrutiny is necessary, as they may affect product texture, flavor, and many other characteristics (Chen et al., 2021). Some studies have indicated that LAB strains such as *Lpb. plantarum* LO3 and *Pediococcus pentosaceus* can impede mold development in different food systems without affecting taste or other physicochemical criteria, which eventually prolongs the storage period (Crowley et al., 2013).

Food preservation techniques can utilize bacterial-enriched edible coatings containing *Lpb. plantarum* to extend the shelf life of fresh produce. These coatings inhibit fungal growth through the production of organic acids. In some strains, such as *Lpb. plantarum*, bacteriocin production may also contribute to antimicrobial activity, creating unfavorable conditions for spoilage microorganisms. Additionally, they help to preserve the physicochemical properties of fruits, such as texture and moisture content, during storage. This approach provides a natural and effective method for enhancing the safety and longevity of perishable goods, reducing spoilage and waste (Agriopoulou et al., 2020a).

### Antifungal activity of LAB in meat products

Undesirable mold control is one of the top priorities for the meat sector, particularly for processed meat. Controlling these undesirable microorganisms will not only ensure the production of safe products but also extend their shelf life while preserving their organoleptic and sensory attributes. In recent years, numerous studies have investigated the antifungal properties of LAB in various processed meat products. Processed meat is a good source of protective LAB in the species such as *Lactococcus lactic*, *Leuconostoc mesenteroides*, *Pediococcus acidilactici*, *Lpb. plantarum*, and *Carnobacterium maltaromaticum*. These LAB strains exhibit antifungal activity through the production of a wide range of antifungal compounds, such as organic acids (e.g., lactic acid), cyclic peptides, reuterin, and other low-molecular-weight bioactive metabolites. While some of these mechanisms have been discussed earlier, they are particularly relevant in meat products, where high moisture and protein levels create an ideal environment for fungal growth. In these systems, the ability of LAB to modify environmental conditions, by lowering pH and reducing water activity, plays a key role in controlling spoilage. For example, LAB can delay mold spoilage in fermented sausages by producing antifungal metabolites, including phenolic acids (such as phenyllactic and benzoic acid) and volatile compounds (such as phenylethyl alcohol and nonanoic acid), which remain active during storage (Nazareth et al., 2023).

LAB have been shown to be effective in managing spoilage and pathogenic fungi in a variety of processed meat products. Álvarez et al. (2020) showed that *Enterococcus faecium* isolated from aged sausage significantly decreased the growth of *Penicillium verrucosum* and *Penicillium nordicum* and its production of mycotoxins under parameters that mimic the ripening process of dry-fermented sausage (Álvarez et al., 2020). *Staphylococcus xylosus,* isolated from the surface of jamón ibérico, inhibited *Penicillium nordicum* growth in dry-cured ham-based medium and reduced ochratoxin A production. An examination led by Zdolec et al. (2008) showed the safety and quality benefits of fermented sausages from Croatia when enhanced with *Latilactobacillus sakei* and mesenterocin. They noted a significant reduction of yeasts without altering the sensory characteristics of the sausage (Zdolec et al., 2008). Najjari et al. (2020) have shown that a mixture of *Latil. sakei* 23 K, *Latil. sakei* BMG95 and *S. xylosus* SUB7037332 XYLO MT111928 was effective for controlling yeast and molds in dry-fermented sausages in Tunisia (Najjari et al., 2020). Significant inhibition of yeast and mold was achieved in vacuum-packaged sliced beef by a combination of *Latil. sakei* CECT4808, and *Latilactobacillus curvatus* CECT 904 (Katikou et al., 2005). The combination of strains can enhance antifungal efficacy through synergistic effects while also improving product quality and shelf life and reduce the risk of resistance in spoilage fungi.

## Conclusion and future directions

The food industry faces ongoing challenges in preserving the quality and safety of perishable food items, while simultaneously prolonging their shelf life. Foods naturally contain microbial communities whose composition is dependent on the nature, origin, and handling or storage conditions of the food. The diversity and complexity of the food microbiome pose significant challenges to the food industry. While food spoilage has been studied for decades, certain microorganisms, such as fungi, particularly those thriving in complex environments or contributing to secondary spoilage through toxin production, were previously underexplored or underestimated in their impact on food safety and quality. Moreover, these microorganisms can evade the traditional preservation methods which primarily target bacterial contamination. However, they are now recognized for their significant role in the rapid spoilage of certain foods. Consequently, the development of new strategies to control fungal contaminants is imperative in the food industry. This urgency is amplified by the increasing consumer demand for natural and safe foods with longer shelf life, as well as the need to reduce food waste and promote sustainable practices in food production and storage.

Studies on the potential of LAB to prevent fungal growth and offsetting mycotoxins have accelerated in the last two decades. However, the antifungal and mycotoxin binding/detoxification properties of LAB isolated from various sources require further investigation.

Discovering LAB strains capable of restraining the growth of mycotoxigenic fungi, particularly those already adapted to specific products, optimizing processing conditions for maximum inhibitory effect, and understanding the molecular mechanisms underlying such inhibition will not only enhance our capacity to produce safer and higher-quality food and feed but also extend their shelf life. Therefore, this will help to reduce significant annual losses in the food industry caused by fungal infestation, contamination, and the presence of mycotoxins. These isolates facilitate multi-step methods for reducing the presence of fungi in the food supply chain. Although no individual isolate or strain can comprehensively address all fungal or food-related challenges, selecting LAB strains that are naturally adapted to the product environment may improve their efficacy. Incorporating such LAB isolates, already recognized as part of the natural microflora in some products, can enhance shelf life, stability, and yield value-added products with a high consumer acceptance rate. However, the success of this new approach remains dependent on several key research and development steps. Future work in this promising field should focus on isolating and characterizing new food-adapted protective cultures with antifungal activity, conducting in-depth structural and functional analyses of antifungal metabolites, and elucidating their mechanisms of action and spectrum of antifungal activity. Additionally, efforts should be directed toward developing eco-friendly and economically viable industrial processes for the large-scale production of novel antifungal ingredients containing protective cultures and/or their active compounds. Finally, large-scale proof-of-concept studies should be conducted to provide more robust scientific data on the effectiveness of these active antifungal ingredients.
